# Kinetics and mechanism of the anilinolysis of aryl phenyl isothiocyanophosphates in acetonitrile

**DOI:** 10.3762/bjoc.9.68

**Published:** 2013-03-26

**Authors:** Hasi Rani Barai, Hai Whang Lee

**Affiliations:** 1 Department of Chemistry, Inha University, Incheon 402-751, Korea

**Keywords:** anilinolysis, cross-interaction constant, deuterium kinetic isotope effects, phosphoryl transfer reaction, reactive intermediates, Y-aryl phenyl isothiocyanophosphates

## Abstract

Kinetic studies on the reactions of Y-aryl phenyl isothiocyanophosphates with substituted X-anilines and deuterated X-anilines were carried out in acetonitrile at 55.0 °C. The free-energy relationships with X in the nucleophiles were biphasic concave upwards with a break region between X = H and 4-Cl, giving unusual positive ρ_X_ and negative β_X_ values with less basic anilines (X = 4-Cl and 3-Cl). A stepwise mechanism with rate-limiting bond breaking for more basic anilines and with rate-limiting bond formation for less basic anilines is proposed based on the positive and negative ρ_XY_ values, respectively. The deuterium kinetic isotope effects involving deuterated anilines (XC_6_H_4_ND_2_) showed primary normal and secondary inverse DKIEs for more basic and less basic anilines, rationalized by frontside attack involving hydrogen-bonded four-center-type TSf and backside attack TSb, respectively. The positive ρ_X_ values with less basic anilines are substantiated by the tight TS, in which the extent of the bond formation is great and the degree of the bond breaking is considerably small.

## Introduction

**Scheme 1:**
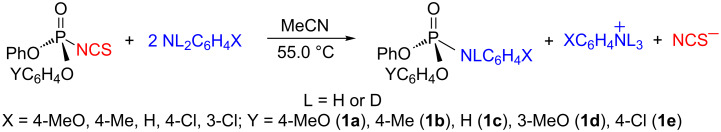
Reactions of Y-aryl phenyl isothiocyanophosphates (**1a**–**e**) with XC_6_H_4_NH_2_(D)_2_ in MeCN at 55.0 °C.

The nucleophilic substitution reactions of tetracoordinate phosphorus have been studied extensively, experimentally and theoretically, in our lab. Two main types of displacement processes are well known in neutral phosphoryl transfer reactions: a stepwise mechanism involving a trigonal bipyramidal pentacoordinate (TBP-5C) intermediate and a concerted displacement at phosphorus through a single pentacoordinate transition state (TS). To extend the kinetic studies on the phosphoryl transfer reactions, the reactions of Y-aryl phenyl isothiocyanophosphates (**1a**–**e**) with substituted anilines (XC_6_H_4_NH_2_) and deuterated anilines (XC_6_H_4_ND_2_) have been investigated kinetically in acetonitrile (MeCN) at 55.0 ± 0.1 °C (Scheme 1). The kinetic results of the present work are discussed based on the selectivity parameters and deuterium kinetic isotope effects (DKIEs). The aim of this work is to gain further information on the substituent effects, DKIEs and mechanism of the phosphoryl transfer reactions.

## Results and Discussion

Table 1 summarizes the second-order rate constants, *k*_H_ and *k*_D_, with X-anilines and deuterated X-anilines, respectively. Table 2 and Table 3 summarize the Hammett (ρ_X(H and D)_) and Brönsted (β_X(H and D)_) coefficients with X, and Hammett coefficients (ρ_Y(H)_) with Y, respectively. The second-order rate constants were obtained from the slopes of pseudo-first-order rate constants (*k*_obsd_) versus aniline concentration (Equation 1). Base-catalysis or noticeable side reactions could be safely ruled out from the zero intercept, *k*_0_ ≈ 0, in MeCN.

[1]
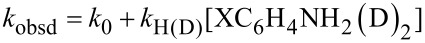


**Table 1:** Second-order rate constants (*k*_(H and D)_ × 10^3^/M^–1^ s^–1^) of the reactions of Y-aryl phenyl isothiocyanophosphates with XC_6_H_4_NH_2_(D_2_) in MeCN at 55.0 °C.

X \ Y		4-MeO (**1a**)	4-Me (**1b**)	H (**1c**)	3-MeO (**1d**)	4-Cl (**1e**)

4-MeO	*k*_H_	9.58 ± 0.02^a^	10.2 ± 0.1	12.7 ± 0.1	17.2 ± 0.1	27.5 ± 0.1
	*k*_D_	7.15 ± 0.04	–	8.92 ± 0.01	–	18.7 ± 0.1
4-Me	*k*_H_	2.71 ± 0.01	3.02 ± 0.01	4.38 ± 0.01	6.09 ± 0.03	9.51 ± 0.01
	*k*_D_	2.30 ± 0.01	–	3.44 ± 0.02	–	7.27 ± 0.05
H	*k*_H_	0.400 ± 0.001	0.517 ± 0.005	0.693 ± 0.004	1.04 ± 0.01	1.89 ± 0.01
	*k*_D_	0.384± 0.001	–	0.647 ± 0.001	–	1.67 ± 0.01
4-Cl	*k*_H_	0.206 ± 0.001	0.257 ± 0.001	0.434 ± 0.001	0.787 ± 0.005	1.67 ± 0.01
	*k*_D_	0.244 ± 0.001	–	0.492 ± 0.001	–	1.81 ± 0.02
3-Cl	*k*_H_	0.718 ± 0.002	0.886 ± 0.008	1.48 ± 0.01	2.68 ± 0.01	5.62 ± 0.03
	*k*_D_	0.994± 0.002	–	2.12 ± 0.01	–	8.46 ± 0.04

^a^Standard deviation.

**Table 2:** Hammett (ρ_X(H and D)_)^a^ and Brönsted (β_X(H and D)_)^b^ coefficients with X for the reactions of Y-aryl phenyl isothiocyanophosphates with XC_6_H_4_NH_2_(D_2_) in MeCN at 55.0 °C.

X \ Y		4-MeO (**1a**)	4-Me (**1b**)	H (**1c**)	3-MeO (**1d**)	4-Cl (**1e**)

4-MeO, 4-Me, H	ρ_X(H)_^c^	−5.09 ± 0.03	−4.76 ± 0.04	−4.68 ± 0.01	−4.51 ± 0.01	−4.29 ± 0.02
	ρ_X(D)_^c^	−4.69 ± 0.02	–	−4.23 ± 0.01	–	−3.87 ± 0.02
	β_X(H)_^c^	1.83 ± 0.08	1.72 ± 0.09	1.69 ± 0.04	1.63 ± 0.04	1.55 ± 0.07
	β_X(D)_^c^	1.69 ± 0.06	–	1.53 ± 0.04	–	1.40 ± 0.06

4-Cl, 3-Cl	ρ_X(H)_	3.87	3.84	3.81	3.80	3.76
	ρ_X(D)_	4.35	–	4.53	–	4.78
	β_X(H)_	−1.18	−1.17	−1.16	−1.16	−1.15
	β_X(D)_	−1.33	–	−1.38	–	−1.46

^a^The σ values were taken from [1]. ^b^The p*K*_a_ values of X-anilines in water were taken from [2]. ^c^Correlation coefficients (*r*) of ρ_X_ and Brönsted β_X_ values for X = (4-MeO, 4-Me, H) are better than 0.996.

**Table 3:** Hammett coefficients (ρ_Y(H)_)^a^ with Y for the reactions of Y-aryl phenyl isothiocyanophosphates with XC_6_H_4_NH_2_ in MeCN at 55.0 °C.

X	4-MeO	4-Me	H	4-Cl	3-Cl

ρ_Y(H)_^b^	0.88 ± 0.07	1.08 ± 0.05	1.27 ± 0.07	1.77 ± 0.09	1.75 ± 0.09

^a^The σ values were taken from [1]. ^b^Correlation coefficients (*r*) of ρ_Y(H)_ values are better than 0.962.

**Figure 1:**
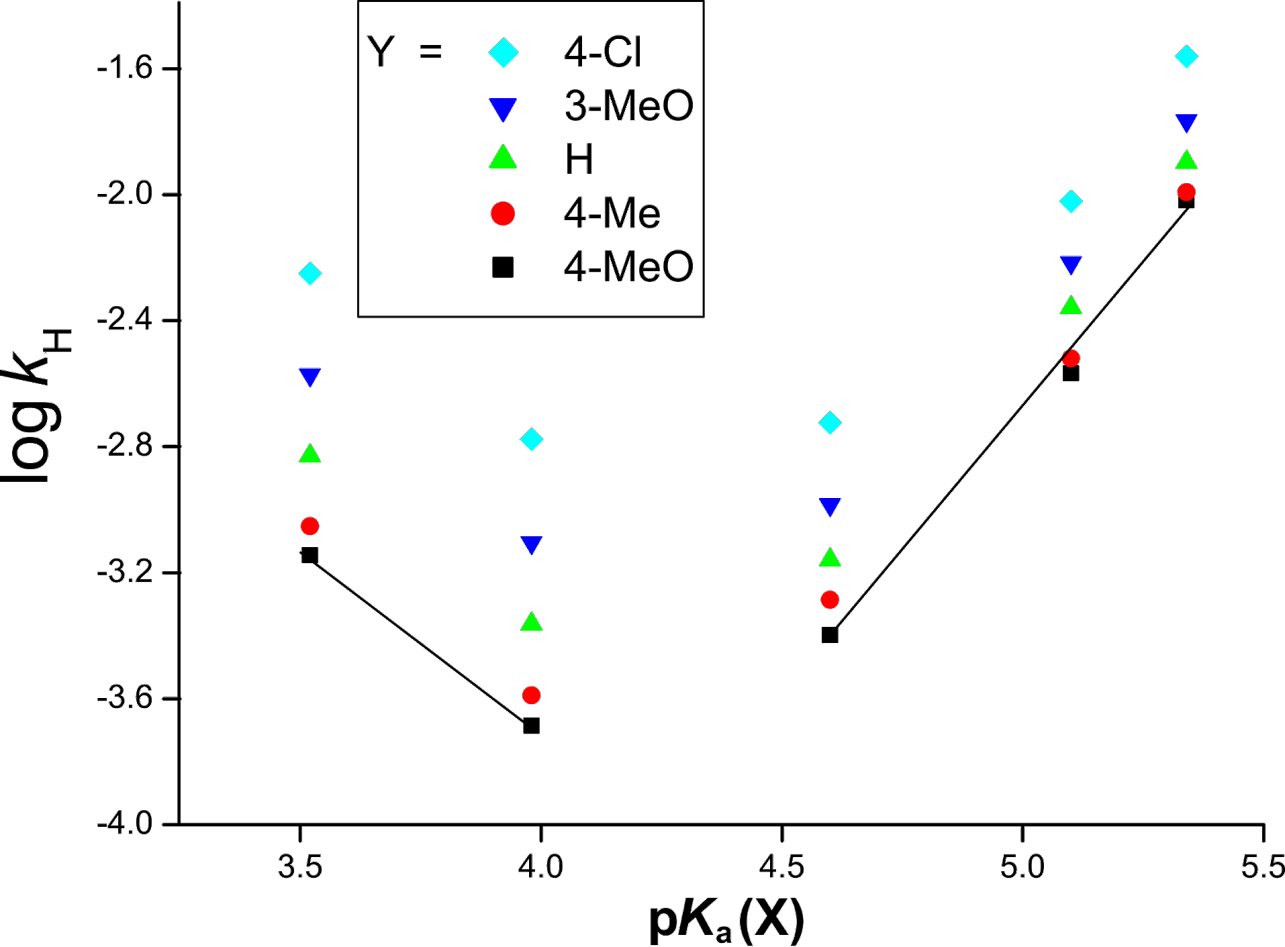
Brönsted plots with X [log *k*_H_ versus p*K*_a_(X)] of the reactions of Y-aryl phenyl isothiocyanophosphates with XC_6_H_4_NH_2_ in MeCN at 55.0 °C.

Figure 1 presents the Brönsted plots with X in the nucleophiles. The substituent effects of X on the reaction rates are not compatible with a typical nucleophilic substitution reaction. The Hammett and Brönsted plots with X are biphasic concave upwards with a break region between X = H and 4-Cl (break point of σ_X_ ≈ 0.13), giving unusual positive ρ_X_ and negative β_X_ values with X = 4-Cl and 3-Cl. Positive ρ_X_ (and negative β_X_) values indicate that the nucleophilic N atom becomes more negative in the TS compared to in the ground state (GS). The substituent effects of Y on the reaction rates are consistent with a typical nucleophilic substitution reaction, and the rate increases with a more electron-withdrawing substituent.

The cross-interaction constant (CIC) is defined based on the substituent effects of the nucleophiles, substrates and/or leaving groups on the reaction rates [3–5]. In the present work, the CIC (ρ_XY_) between substituents X and Y in the nucleophiles and substrates, respectively, is described in Equation 2 and Equation 3.

[2]
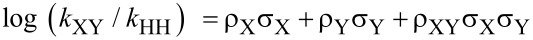


[3]
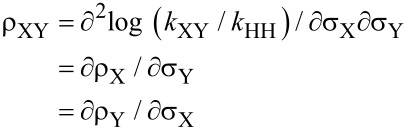


**Figure 2:**
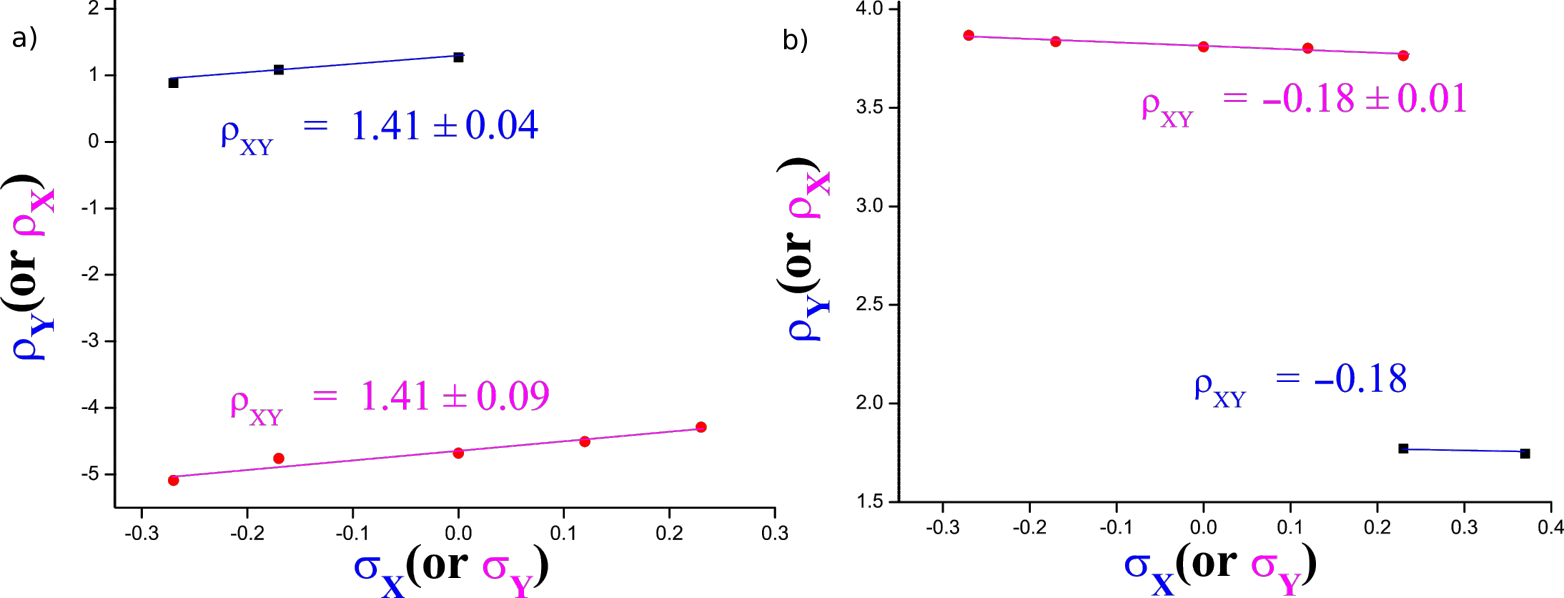
Plots of ρ_X_ versus σ_Y_ and ρ_Y_ versus σ_X_ of the reactions of Y-aryl phenyl isothiocyanophosphates with XC_6_H_4_NH_2_ in MeCN at 55.0 °C. The obtained ρ_XY_ values by multiple regression are as follows: (a) ρ_XY_ = 1.40 ± 0.06 (*r* = 0.992) with more basic anilines and (b) ρ_XY_ = –0.18 ± 0.09 (*r* = 0.975) with less basic anilines.

The two ρ_XY_ values are obtained because the Hammett plots with X are biphasic. Figure 2 shows the plots of ρ_X_ versus σ_Y_ and ρ_Y_ versus σ_X_ to determine the ρ_XY_ values, according to Equation 3. The signs of ρ_XY_ are positive with more basic anilines (X = 4-MeO, 4-Me, H) but negative with less basic anilines (X = 4-Cl, 3-Cl). The number of kinetic data points, 10 *k*_H_ values with less basic anilines, to obtain the ρ_XY_ value is not enough to overcome the experimental error. At least, however, the sign of ρ_XY_ is acceptable, and the greater magnitude of ρ_XY_ value with more basic anilines than that with less basic anilines is also acceptable. Accordingly, the authors propose the following reaction mechanism: (i) a stepwise process with rate-limiting leaving-group departure from the intermediate for more basic anilines based on the positive sign of ρ_XY_ (= 1.40), and (ii) a stepwise process with rate-limiting bond formation for less basic anilines based on the negative sign of ρ_XY_ (= −0.18) [3–5]. The greater magnitude of ρ_XY_ value with more basic anilines compared to that with less basic anilines suggests that the interaction between X and Y with more basic anilines is larger than that with less basic anilines in the TS (see below).

**Scheme 2:**
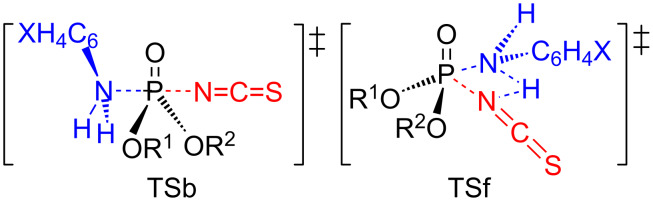
Backside attack involving in-line-type TSb and frontside attack involving a hydrogen-bonded, four-center-type TSf.

The DKIEs are primary normal with more basic anilines (X = 4-MeO, 4-Me, H) while secondary inverse with less basic anilines (X = 4-Cl, 3-Cl) as shown in Table 4. Primary normal DKIEs (*k*_H_/*k*_D_ > 1.0) indicate that partial deprotonation of the aniline occurs in a rate-limiting step by hydrogen bonding [6–12]. In contrast, secondary inverse DKIEs (*k*_H_/*k*_D_ < 1.0) indicate that the N–H(D) vibrational frequencies invariably increase upon going to the TS because of an increase in the steric congestion in the bond-making process in a stepwise process with a rate-limiting bond formation (or a normal S_N_2 reaction) [13–15]. The magnitudes of the *k*_H_/*k*_D_ values invariably decrease as the aniline becomes less basic. The magnitudes of the *k*_H_/*k*_D_ values invariably increase for X = (4-MeO, 4-Me, H, 4-Cl) while they invariably decrease for X = 3-Cl as the substituent Y changes from electron-donating to electron-withdrawing. The maximum value of *k*_H_/*k*_D_ = 1.47 with X = 4-MeO and Y = 4-Cl indicates extensive hydrogen bonding whereas the minimum value of *k*_H_/*k*_D_ = 0.66 with X = 3-Cl and Y = 4-Cl indicates severe steric congestion in the TS, suggesting a great extent of bond formation. The secondary inverse and primary normal DKIEs are substantiated by backside nucleophilic attack involving in-line-type TSb and frontside attack involving hydrogen-bonded, four-center-type TSf, respectively (Scheme 2).

**Table 4:** The DKIEs (*k*_H_/*k*_D_) of the reactions of Y-aryl phenyl isothiocyanophosphates with XC_6_H_4_NH(D)_2_ in MeCN at 55.0 °C.

X \ Y	4-MeO (**1a**)	H (**1c**)	4-Cl (**1e**)

4-MeO	1.34 ± 0.01^a^	1.42 ± 0.01	1.47 ± 0.01
4-Me	1.18 ± 0.01	1.27 ± 0.01	1.31 ± 0.01
H	1.04 ± 0.01	1.07 ± 0.01	1.13 ± 0.01
4-Cl	0.844 ± 0.005	0.882 ± 0.003	0.922 ± 0.012
3-Cl	0.722 ± 0.002	0.698 ± 0.001	0.664 ± 0.004

^a^Standard error {= 1/*k*_D_[(^Δ^*k*_H_)^2^ + (*k*_H_/*k*_D_)^2^ × (^Δ^*k*_D_)^2^]^1/2^} from [16].

**Scheme 3:**
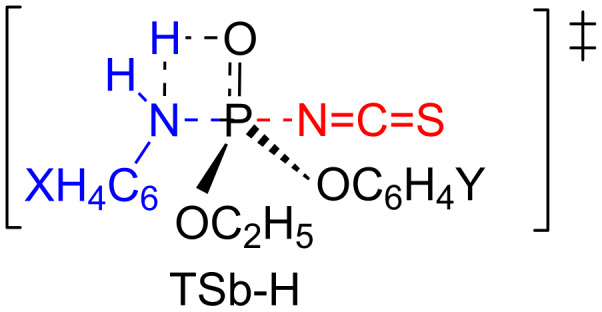
Backside attack involving a hydrogen-bonded, four-center-type TSb-H.

The hydrogen-bonded four-center type of TSb-H could be another plausible TS structure, in which hydrogen bonding of an amine hydrogen atom occurs on the P=O oxygen atom (Scheme 3). In the present work, three possible TSs could substantiate the primary normal DKIEs: (i) TSf, (ii) TSb-H or (iii) both TSb-H and TSf. The anilinolyses of tetracoordinate phosphorus with the Cl^–^ leaving group have been extensively studied in this lab, and the obtained data of primary normal DKIEs involving deuterated anilines are rationalized by TSf-type in which hydrogen bonding of an amine hydrogen atom occurs on the departing chloride [17–23]. The authors also suggested TSf-type, in which hydrogen bonding of an amine hydrogen atom occurs to the departing phenoxy oxygen atom for the anilinolyses of aryl dimethyl, methyl and diphenyl phosphinates [24]. Thus, at this point, the authors are in favor of TSf for the present work.

The focus will now shift to the unusual positive ρ_X_ and negative β_X_ values with X = 4-Cl and 3-Cl. These values can be observed because of (i) desolvation of the GS [25–26] or (ii) TS imbalance phenomenon [27–29]. However, in the present work, the positive ρ_X_ and negative β_X_ values for less basic anilines are not ascribed to (i) a desolvation step prior to the rate-limiting nucleophilic attack, because the aniline nucleophile is neutral and the MeCN solvent is dipolar aprotic; and to (ii) a TS imbalance phenomenon, because the leaving group of isothiocyanate is too poor to form an ion-pair type. The unusual positive ρ_X_ (and negative β_X_) values with X = (4-Cl and 3-Cl) indicate that the nucleophilic N atom becomes more negative in the TS compared to in the GS. The authors, thus, propose that the structure of the TS is similar to what would be if the isothiocyante were the nucleophile and aniline were the leaving group, such that the nucleophilic N atom becomes more negative in the TS compared to in the GS. In other words, the TS is very tight and in which the degree of bond formation is very great while the degree of bond breaking is considerably small, resulting in the positive ρ_X_. The very small value of *k*_H_/*k*_D_ = 0.66 with X = 3-Cl and Y = 4-Cl could be supporting evidence. The relatively small magnitude of ρ_XY_ = −0.18 with less basic anilines could be another piece of supporting evidence, because the normal S_N_2 mechanism (or stepwise mechanism with rate-limiting bond formation) gives a magnitude of ρ_XY_ ≈ −0.7 [3–5]. This would be attributed to the strong interaction between the nucleophile (X) and leaving group over the nucleophile (X) and substrate (Y) (see above).

Finally, the authors propose the following reaction mechanism of the present work: (i) for more basic anilines (X = 4-MeO, 4-Me, H), a stepwise process with rate-limiting leaving-group departure from the intermediate, involving a predominant frontside attack with a hydrogen-bonded four-center-type TSf based on the positive ρ_XY_ and primary normal DKIEs; and (ii) for less basic anilines (X = 4-Cl , 3-Cl), a stepwise process with rate-limiting bond formation, involving a predominant backside attack TSb, and very tight TS, in which the extent of the bond formation is great and the degree of bond breaking is very small based on the negative ρ_XY_, secondary inverse DKIEs and positive ρ_X_.

Activation parameters, enthalpies and entropies of activation, are determined as shown in Table 5. The enthalpies of activation are relatively low and entropies of activation are of relatively large negative value. The relatively low value of activation enthalpy and large negative value of activation entropy are typical for the aminolyses of P=O systems, regardless of the mechanism, whether stepwise with rate-limiting bond formation (or a concerted) or stepwise with rate-limiting bond breaking.

**Table 5:** Activation parameters for the reactions of Y-aryl phenyl isothiocyanophosphate with aniline (C_6_H_5_NH_2_) in MeCN.

Y	*t*/°C	*k*_H_ × 10^4^/M^−1^ s^−1^	∆*H^≠^*/kcal mol^−1^	−∆*S^≠^*/cal mol^−1^* K*^−1^

4-MeO (**1a**)	45.0	2.91 ± 0.01	5.7 ± 0.1	56 ± 1
	55.0	4.00 ± 0.01		
	65.0	5.29 ± 0.07		
4-Me (**1b**)	45.0	3.72 ± 0.01	6.2 ± 0.1	55 ± 1
	55.0	5.17 ± 0.05		
	65.0	7.08 ± 0.01		
H (**1c**)	45.0	4.96 ± 0.01	6.3 ± 0.1	54 ± 1
	55.0	6.93 ± 0.01		
	65.0	9.51 ± 0.01		
3-MeO (**1d**)	45.0	7.24 ± 0.01	6.2 ± 0.4	54 ± 1
	55.0	10.4 ± 0.1		
	65.0	13.7 ± 0.1		
4-Cl (**1e**)	45.0	13.5 ± 0.1	6.1 ± 0.1	53 ± 1
	55.0	18.9 ± 0.1		
	65.0	25.5 ± 0.2		

## Experimental

**Materials**. HPLC grade acetonitrile (water content is less than 0.005%) was used without further purification. Deuterated anilines were synthesized as previously described [17–24]. Substrates were prepared as described earlier [30].

**Kinetics measurement**. Rates were measured conductometrically at 55.0 °C as described previously [17–24]. The initial concentrations of substrates and nucleophiles were as follows; [substrate] = 5 × 10^−3^ M and [X-aniline] = 0.10–0.30 M. The second-order rate constants (*k*_H(D)_) were determined for at least five concentrations of anilines. The *k*_obsd_ values were the average of at least three runs.

**Product analysis**. Diphenyl isothiocyanophosphate was reacted with excess aniline for more than 15 half-lives at 55.0 °C in MeCN. Acetonitrile was evaporated under reduced pressure. The product mixture was treated with ether by a work-up process with dilute HCl and dried over anhydrous MgSO_4_. The product was isolated through column chromatography (30% ethyl acetate/*n*-hexane) and then dried under reduced pressure. The analytical and spectroscopic data of the product gave the following results (see also Supporting Information File 1):

**[(C_6_H_5_O)_2_P(=O)NHC_6_H_5_].** White solid crystal; mp 132–133 °C; ^1^H NMR (400 MHz, MeCN-*d*_3_) δ 6.66 (br d, *J* = 8.8 Hz, 1H, aliphatic), 7.01–7.43 (m, 15H, aromatic); ^13^C NMR (100 MHz, MeCN-*d*_3_) δ 118.59–131.20 (m, 18C; aromatic); ^31^P NMR (162 MHz, MeCN-*d*_3_) δ 3.62 (d, *J* = 8.6 Hz, 1P, P=O); GC–MS (EI, *m*/*z*): 325 (M^+^).

## Supporting Information

File 1 Spectra of product.
