# Supplementary material for: Kinetics and mechanism of the anilinolysis of aryl phenyl isothiocyanophosphates in acetonitrile
Source: Beilstein J Org Chem. 2013 Mar 26;9:615–20. doi: 10.3762/bjoc.9.68 (PMC3628914; doi:10.3762/bjoc.9.68)
Supplement: File 1 — Spectra of product. [file Beilstein_J_Org_Chem-09-615-s001.pdf]

Supporting Information

for

Kinetics and mechanism of the anilinolysis of aryl phenyl isothiocyanophosphates in acetonitrile

Hasi Rani Barai and Hai Whang Lee*

Address: Department of Chemistry, Inha University, Incheon 402-751, Korea

Email: Hai Whang Lee* - hwlee@inha.ac.kr

* Corresponding author

Spectra of product

Product: [$(C_6H_5O)_2P(=O)NHC_6H_5$]

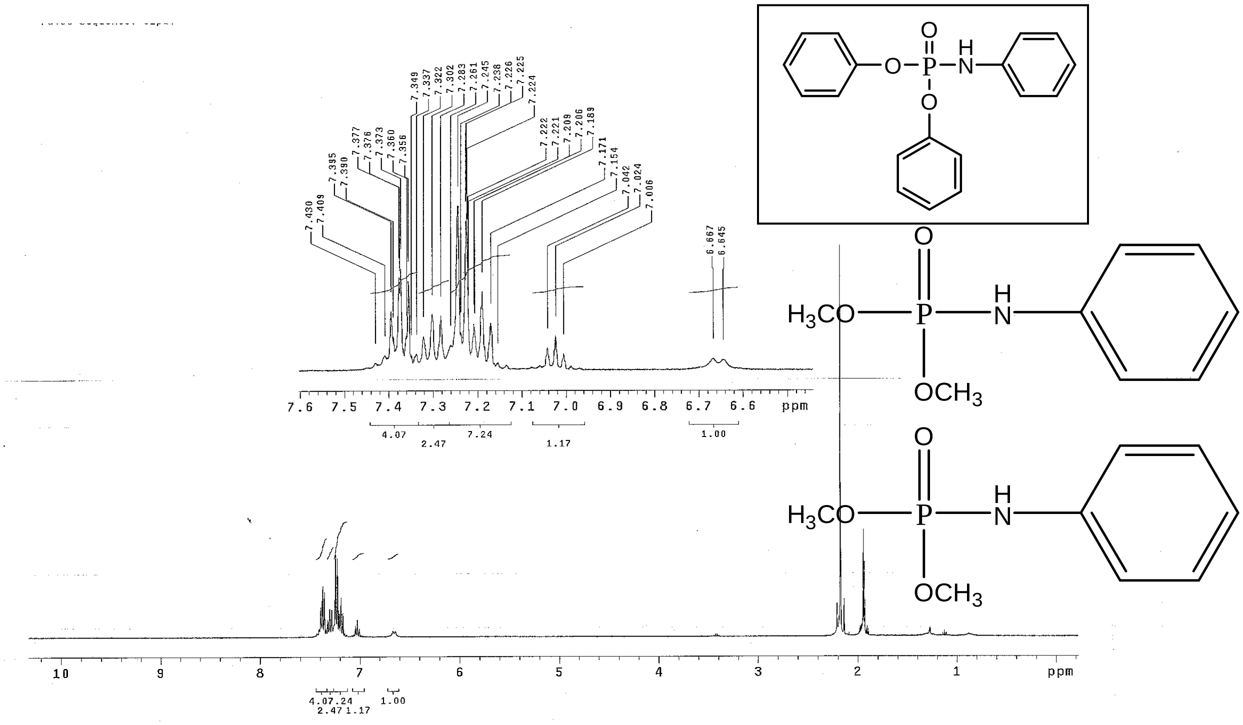

Figure S1: The ^{1}H NMR spectrum of [$(C_6H_5O)_2P(=O)NHC_6H_5$].

Product: [$(C_6H_5O)_2P(=O)NHC_6H_5$]

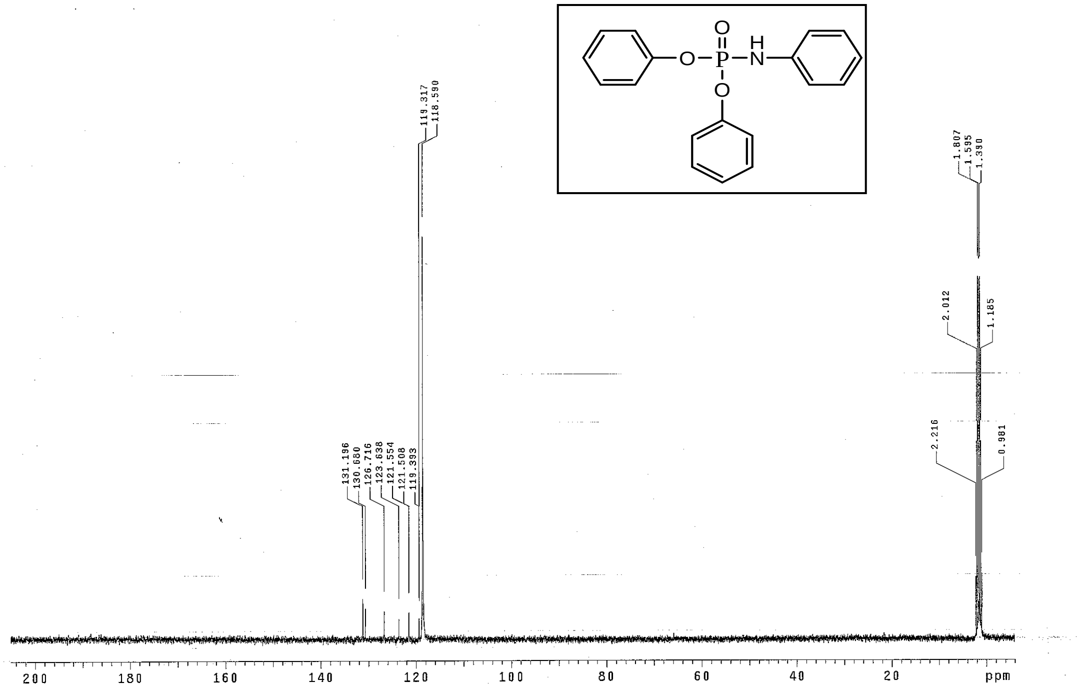

Figure S2: The ^{13}C NMR spectrum of [$(C_6H_5O)_2P(=O)NHC_6H_5$].

Product: [$(C_6H_5O)_2P(=O)NHC_6H_5$]

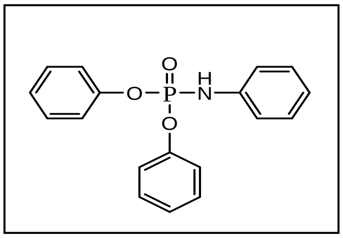

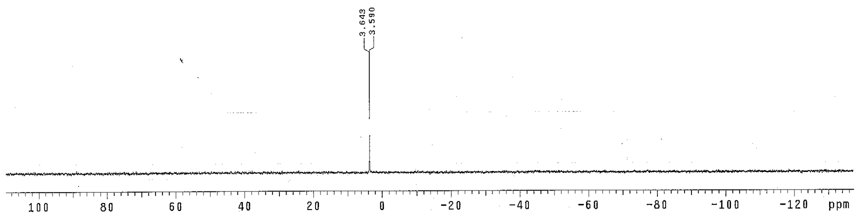

Figure S3: The ^{31}P NMR spectrum of [$(C_6H_5O)_2P(=O)NHC_6H_5$].

Product: [$(C_6H_5O)_2P(=O)NHC_6H_5$]

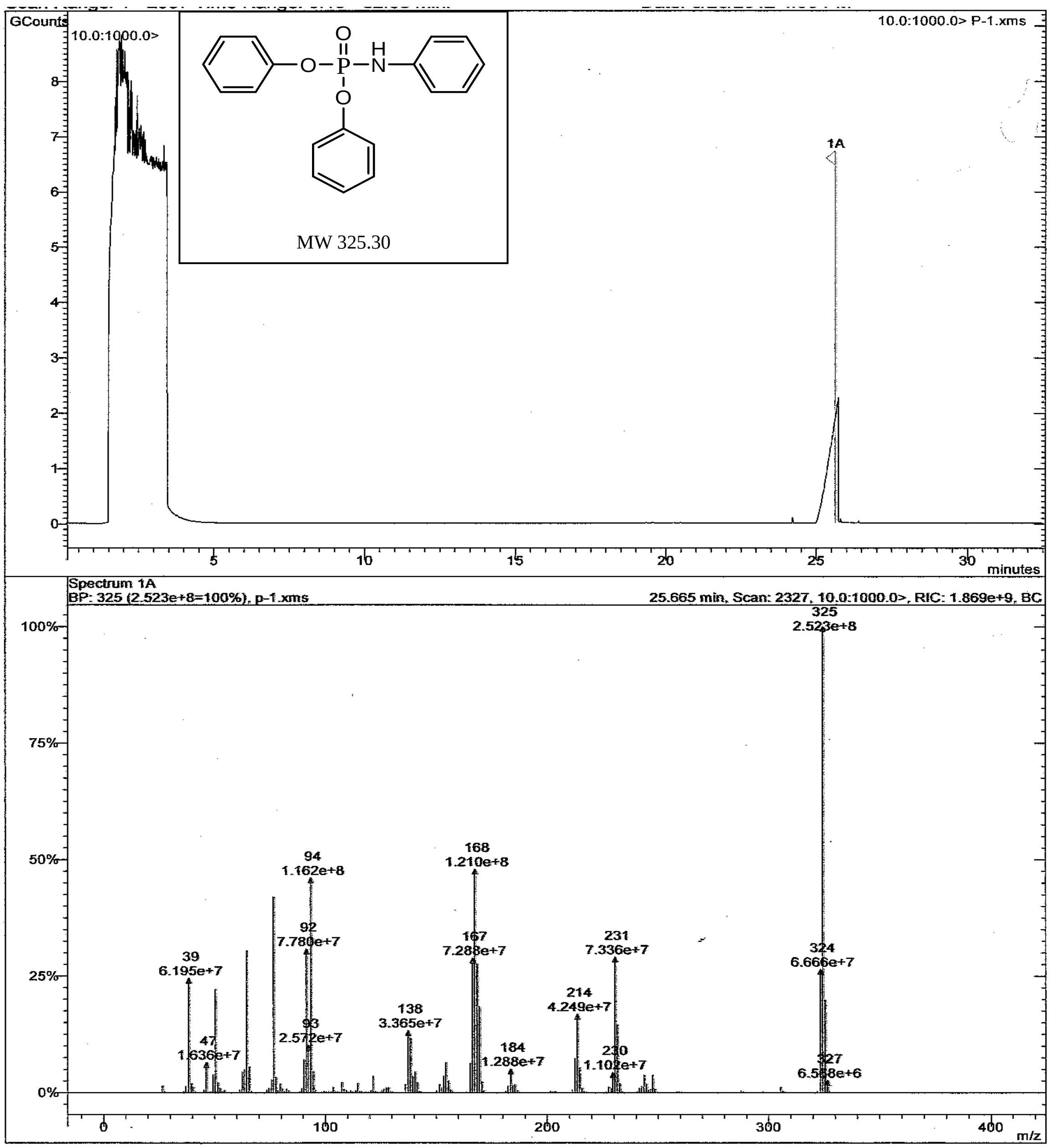

Figure S4: The GC–MS spectrum of [$(C_6H_5O)_2P(=O)NHC_6H_5$].
